# Age Differences in the Experience of Daily Life Events: A Study Based on the Social Goals Perspective

**DOI:** 10.3389/fpsyg.2017.01623

**Published:** 2017-09-20

**Authors:** Lingling Ji, Huamao Peng, Xiaotong Xue

**Affiliations:** ^1^Institute of Developmental Psychology, Beijing Key Laboratory of Applied Experimental Psychology, Beijing Normal University Beijing, China; ^2^Faculty of Education, Beijing City University Beijing, China; ^3^Qin’an Road Primary School Lanzhou, China

**Keywords:** daily events, social goals, emotional experience, daily diary method, positivity effect

## Abstract

This study examined age differences in daily life events related to different types of social goals based on the socioemotional selectivity theory (SST), and determined whether the positivity effect existed in the context of social goals in older adults’ daily lives. Over a course of 14 days, 49 older adults and 36 younger adults wrote about up to three life events daily and rated the valence of each event. The findings indicated that (1) although both older and younger adults recorded events related to both emotional and knowledge-acquisition goals, the odds ratio for reporting a higher number of events related to emotional goals compared to the number of events related to knowledge-acquisition goals was 2.12 times higher in older adults than that observed in younger adults. (2) Considering the number of events, there was an age-related positivity effect only for knowledge-related goals, and (3) older adults’ ratings for events related to emotional and knowledge-acquisition goals were significantly more positive compared to those observed in younger adults. These findings supported the SST, and to some extent, the positivity effect was demonstrated in the context of social goals.

## Introduction

According to the socioemotional selectivity theory [(SST) Carstensen et al., 1999], people’s perceptions of a future time in their lives influence their motivation and goal preference. The SST focuses on two main classes of social goals. According to the SST, people’s social goals can be categorized as emotional or knowledge-acquisition goals (Carstensen, 1992; Carstensen et al., 1999, 2003). Emotional goals or present-focused goals are concerned with an individual’s experiences of different types of emotions, regulation of emotions, and sensing others’ emotions during the process of social interaction. Knowledge-acquisition or future-oriented goals concern individuals’ need to acquire information or knowledge, make new social contacts, and expand their horizons. According to the theory, knowledge and emotion related goals comprise an essential constellation of goals that motivate social behavior throughout life.

### Age Differences in Social Goals

The prioritization of these two types of goals depends on one’s perception of the future. As older adults perceive time to be limited, they prefer being with familiar social partners, improving intimacy, acquiring emotional satisfaction, and pursuing emotionally meaningful goals during their time. This population generally tends to prioritize emotional goals (Fredrickson and Carstensen, 1990; Carstensen et al., 2003; Mather and Carstensen, 2005). Emotional goals concern an individual’s experiences of different types of emotions, regulation of emotions, and sensing others’ emotions during the process of social interaction. Fung et al. (1999) provided participants (aged 8–93 years) with one familiar partner and two new partners and presented two scenarios in which participants were required to associate with one of the partners; the results indicated that older participants preferred the familiar social partner. In addition, Tan (2011, Unpublished) analyzed differences in life goals between individuals who participated in different types of activities and found that development- and emotion-related goals, rather than goals involving improvements in physical health, were indicators of activity in elderly individuals. In contrast, younger adults, who perceived the future as infinite and had ample time to achieve their goals, engaged in new experiences and sought to broaden their knowledge. They tend to prioritize knowledge-acquisition goals. Knowledge-acquisition goals concern an individual’s need to acquire information or knowledge, make new social contacts, and expand their horizons. The priorities reflected by social goals differ according to age. Does this indicate that older adults are more concerned about events related to emotion, rather than knowledge-acquisition, in daily life, while the opposite is true for younger adults? According to the SST, when knowledge-acquisition goals compete with goals involving emotion regulation, the relative importance of the two goals is measured, and action is taken accordingly (Carstensen et al., 1999). However, competition between the two social goals might not always occur in real life. Thus, whether older and younger adults show different priorities of two social goals in daily life events is the first question in which we are interested. That is to say, whether older adults prioritize daily life events related to emotional goals and younger adults prioritize daily life events related to knowledge-acquisition goals.

### Positivity Effects of Different Social-Goal Contexts

Because older adults are particularly strongly motivated by goals related to emotional satisfaction, the SST predicts an information-processing shift toward a need for positive information in later life (Reed et al., 2014). Age-related differences in the processing of emotionally valenced information (i.e., a preferential shift toward positive information) were first considered within the context of the SST (Carstensen, 2006). The term “positivity effect” refers to an age-related increase in preference for positive information, rather than negative, in attention and memory (Carstensen and Mikels, 2005; Mather and Carstensen, 2005).

According to the SST, the age-related positivity effect could be elicited by emotional goals. However, the age-related positivity effect may also be involved in the process of knowledge-acquisition goals. Most studies examining the positivity effect have been conducted within the theoretical context of the SST using laboratory experiments and word picture materials to explore the positivity effect in attention and memory (Mather and Carstensen, 2005; Isaacowitz et al., 2006; Shamaskin et al., 2010; Isaacowitz and Blanchard-Fields, 2012; Rovenpor et al., 2013). However, social goals were not included as variables in these studies. Whether the positivity effect exists in events related to goals involving knowledge acquisition remains unclear. We assumed that the positivity effect would differ according to social goals and sought to determine the importance of social context (Aldao, 2013). Older adults could pay more attention to emotion-related events in daily life, and their emotional experiences are more positive relative to those of younger adults. In addition, although events related to knowledge do not occur frequently in their daily lives, older adults could remember the positive aspects of this type of event. Therefore, the positivity effect could both be reflected in events related to emotional and knowledge-acquisition goals.

Laboratory experiments included in previous studies have tended to rely on weak emotion elicitors such as synthetic face stimuli and word lists, and were unlikely to alter emotional states regardless of processing tendencies. Moreover, researchers have generally induced emotions using films or photographs under controlled situations. However, these situations might not reflect those experienced by older adults in daily life. Sims et al. (2015) proposed that findings from laboratory studies could reflect laboratory-based experiments’ failure to capture the regulatory strategies that older people use in their everyday lives. Therefore, whether the positivity effect exists in older adults’ daily life in the context of social goals is the second question of the present study. Thus, we investigated whether there was evidence of the positivity effect when pursuing daily emotional and knowledge-acquisition goals.

A daily diary method was used in the study. This is the most commonly used method for examining daily emotional experiences that could enhance our understanding of participants’ daily activities, social interactions, and moods (Reis et al., 2000; Bylsma et al., 2011; Conner and Mehl, 2015; Conner and Silvia, 2015). For example, Conner and Silvia (2015) used an Internet daily diary method to understand the ecology of participants’ daily creativity, how certain emotions may help or hinder creative pursuits, and who behaves more creatively. The method has ecological validity and emphasizes the status of things, the relation between variables in the natural state, or a situation with which individuals are familiar; and generating more real and believable thoughts, emotions, and behavior (Hormuth, 1986). Moreover, the daily diary method can document participants’ activities since participants’ social goals may be embodied in these activities (Hormuth, 1986).

Therefore, the objectives of our study were as follows: firstly, explore whether older and younger adults showed different priorities toward social goals in daily life events. Secondly, assess whether older adults recorded a higher number of positive events than negative events when compared to younger adults with respect to emotional and knowledge-acquisition goals. Thirdly, evaluate whether older adults’ ratings for events related to emotional and knowledge-acquisition goals were significantly more positive compared to those observed in younger adults.

## Materials and Methods

### Participants

There were 85 participants in our study. Forty-nine older adults (age range 56–72 years, *M* = 63.80, *SD* = 3.60 years) were recruited from a community for the elderly in Beijing and 36 younger adults (age range: 20–28 years, *M* = 22.94, *SD* = 2.14 years) were recruited from Beijing Normal University. The mean value of years of education reported by the older and younger groups were 12.88 (*SD* = 2.40) and 16.42 (*SD* = 1.03), respectively. Since we focused on the content of participants’ diaries, and not on their language or written skills, their ability with respect to vocabulary was not measured.

To conduct a power analysis, the effect size of previous studies was used. The positivity effect meta-analysis by Reed et al. (2014) stated that the effect size among unconstrained studies was 0.482. Unconstrained studies did not constrain information processing through instructional manipulations and/or task-related restrictions, but afforded and encouraged naturalistic and unconstrained processing of stimuli, which our study belongs to. Thus, when the sample size was 85 and the effect size was 0.482; the result of *GPower* showed that the present study’s statistical power reached 0.86.

### Data Collection

The participants were instructed to record daily events for 14 days. Each participant was provided with a diary notebook that included the following instructions on the first page:

“This is a study examining daily life events and will last 2 weeks. You should spend only 10 min per day on this task. Because our study is anonymous, you can record real life events. Please write about up to three events that made you feel happy (warm, excited, comfortable, or relaxed) or unhappy (impatient, depressed, disturbed, angry, or tired) before going to sleep each day. After writing about each event, please rate your feelings regarding the event using one of the numbers in the following guide: 1 = very negative, 2 = more negative, 3 = neutral, 4 = more positive, 5 = very positive. Thank you for your cooperation.”

Reminders were sent to older adults through text messages, and younger adults were notified through WeChat, an instant messaging service. Reminders were sent periodically to maximize compliance and ensure that the participants do not experience any difficulty in completing the task. In addition, participants were instructed to rate the valence of the life events that reflected their emotional experiences. Older adults came to our laboratory to return the diaries after 2 weeks, and younger adults sent their diary entries via email once a week.

As for events valence, participants were asked to rate the valence of each event that they had recorded, using a scale ranging from 1 (very negative) to 5 (very positive). Based on participants’ evaluations of their event-induced emotional experiences, events rated 5 (very positive) or 4 (more positive) were labeled “positive events,” events rated 3 (neutral) were labeled “neutral events,” and events rated 2 (more negative) or 1 (very negative) were labeled “negative events.”

### Coding System

The development of the entire coding system involved four stages. The first stage included open coding. During this stage, five diaries, which included approximately 100 events, were selected to identify key indicators (e.g., purpose, actions, emotions, and words) related to two social goals. These indicators were then clustered into several categories according to the definitions of knowledge-acquisition and emotional goals (Carstensen, 1992; Carstensen et al., 1999, 2003). At the end of the first stage, rough criteria were established for events related to the two types of social goals.

The second stage involved constant comparisons. The remaining diaries were compared against the existing rough criteria, and new criteria were added as necessary. When no new criteria emerged, the comparison was considered complete.

The third stage was to determine time orientation. As the key to distinguish between the two social goals was time orientation (Carstensen et al., 1999), the most important criterion in our coding system was to determine whether the events reported focused on preparation for the future or satisfaction with the present. Therefore, we sought to establish a definition of “future” according to the temporal distance between the present and future in participants’ minds. A pilot survey was conducted to establish a definition for “future.” The survey was conducted with 18 older adults (aged 63–77 years) and 30 younger adults (aged 19–25 years), and the results showed that the majority of participants considered 6 months later to be the future. Therefore, events involving a point in time at least 6 months later were coded as events related to knowledge-acquisition goals, and those involving a point in time within 6 months were coded as events related to emotional goals.

The fourth stage involved assessing the validity and reliability of the coding system. To assess the validity of the coding system, we invited five experts (one postdoctoral researcher and one doctoral researcher from Carstensen’s lab, a doctoral researcher from the Chinese University of Hong Kong, an assistant professor from the Education University of Hong Kong, and a psychology professor from Beijing Normal University) with experience in the SST research to rate the degree to which each individual coding criterion and all of the statements combined were consistent with the operational definitions of the goal types using a 7-point scale ranging from 1 (not at all) to 7 (extremely). The experts’ average rating for all criteria for events related to both types of goals was 6.14 (*SD* = 0.66) that indicated that the coding system demonstrated a high level of validity. Two undergraduate students and one graduate student, who were unfamiliar with the study, were recruited as raters and coded the diaries independently. The interrater reliability result (Kendall’s *W* = 0.96) confirmed that the coding system was reliable. All events were classified as events related to knowledge-acquisition, emotional, or mixed goals, using the coding system. Events were classified as related to knowledge-acquisition goals if their content fulfilled any of the following criteria and did not conflict with the criteria for emotional goals: (a) knowledge-acquisition goals related to the future (if an event referred to words related to time, such as 6 months later, or in the future; the event was classified as related to knowledge-acquisition goals; there was a wide range of non-future events which were coded as present-focused events); (b) knowledge acquisition; (c) an individual’s observation, understanding, and learning of social survival skills through interaction with others, and inspiration for knowledge acquisition from the people around them, events, and their own life experiences; (d) realization of one’s own shortcomings through interaction with others and engagement in periodic self-assessment; and (e) use of activities and events to provide information (reading books, etc.). Events were classified as related to emotional goals if their content fulfilled any of the following criteria and did not conflict with the criteria for knowledge-acquisition goals: (a) emotional goals related to the present (if the event referred to words related to time, such as now, the present, and were within 6 months; the event was classified as related to emotional goals); (b) mood control rather than pursuit of new knowledge (emotion can be affected by tourism, scenery, environment, weather, prices, or physical conditions, and individuals can acquire emotional experience and become inspired by events and the people around them); (c) preference for spending time with family and close friends, rather than going to a party to obtain emotional satisfaction (sometimes, individuals need to feel needed and supported by others, and people acquire positive emotional experience by helping others); (d) a focus on the meaning of life and emotional intimacy (individual’s might seek to control their own moods or to engender a positive emotional experience); and (e) a focus on emotional rewards achieved through various activities or participation in social activities for the sake of an emotional experience. If an event could be classified as related to either an emotional or a knowledge-acquisition goal; it was classified as a mixed event. In particular, if an event could be classified as related to knowledge-acquisition goals or emotional goals according to its contents, then the time orientation (i.e., 6-month) that it referred to was not considered. The details of the coding system are shown in **Table 1**.

**Table 1:** Detailed instructions of the coding system.

(1) Events were classified as related to knowledge-acquisition goals if their content fulfilled any of the following criteria and did not conflict	
with the criteria for emotional goals.	
(a) Knowledge-acquisition goals related to the future (if an event referred to words related to time, such as 6 months later, or in the future; the event was classified as related to knowledge-acquisition goals. There was also a wide range of non-future events which were coded as present-focused events).	e.g., “The problems in middle school math competitions became increasingly difficult in today’s classes, which stumped a number of my classmates. Only three questions were covered in the two classes, and I did still not understand the problem-solving process. I was very concerned about my future studies.”

(b) Knowledge acquisition	e.g., “This morning, I attended a project in which student volunteers taught us how to use computers face-to-face at the University of Posts and Telecommunications. The two problems that I didn’t understand before were solved. Thus, I was very happy.”

(c) An individual’s observation, understanding; learning of social survival skills through interaction with others; and inspiration for knowledge acquisition from the people around them, events, and their own life experiences.	e.g., “Today, I went to the Aging Lab at Beijing Normal University to take part in tests. It was a very happy thing and I acquired much knowledge constantly. It’s never too late to learn.”

(d) Realization of one’s own shortcomings through interaction with others and engagement in periodic self-assessment.	e.g., “The lesson on the Study on New Venture’s Creation was boring. The teacher suddenly asked, ‘What are you interested in? What do you care about?’ Then, I realized that I did not know how to answer these questions. I have spent days and months without doing anything meaningful. While listening to classmates’ answers, I was lost in thought.”

(e) Use of activities and events to provide information (reading books, etc.)	e.g., “On Friday evening, I turned on the computer and watched *Tian tian Xiang shang*, a famous, entertaining talk-show in China. The hosts sounded very happy. I should imitate their speech to improve my public-speaking skills.”

**(2) Events were classified as related to emotional goals if their content fulfilled any of the following criteria and did not conflict**	
**with the criteria for knowledge-acquisition goals.**	

(a) Emotional goals related to the present (if the event referred to words related to time, such as now, the present, and were within 6 months; the event was classified as related to emotional goals).	e.g., “On Sunday, my husband’s colleague, whom we had not seen for many years, was coming. We were all happy. They were very excited when they chatted about their time at the company.”

(b) Mood control rather than pursuit of new knowledge (emotion can be affected by tourism, scenery, environment, weather, prices, or physical conditions, and individuals can acquire emotional experience and become inspired by events and the people around them).	e.g., “This morning, when I pulled back the curtain, a silver world appeared outside the window! Snow was everywhere, just lying on the trees. An unseasonable snowfall gave a surprise to spring. Particularly in these days of the hazy weather, the snow scene was a rare and beautiful thing.”

(c) Preference for spending time with family and close friends, rather than going to a party to obtain emotional satisfaction (sometimes, individuals need to feel needed and supported by others, and people acquire positive emotional experience by helping others).	e.g., “On the way to the library, a man asked me how to get to the classroom. I explained the way to him clearly and patiently. I had a strong feeling of presence and being needed as soon as he thanked me, which made me very happy.”

(d) A focus on the meaning of life and emotional intimacy (individual’s might seek to control their own moods or to engender a positive emotional experience).	e.g., “Although it was late, I still went for a walk with my friend. After I poured out my problems to her, I felt quite relaxed. She calmed me down and let me think about some things objectively. I thanked her a lot.”

(e) A focus on emotional rewards achieved through various activities or participation in social activities for the sake of an emotional experience.	e.g., “I had a good time with my friends at the beginning of this morning’s exercise. The teacher who taught us to make fans came to the classroom at 9:00. Some of her behaviors made us feel uncomfortable. I attended this class for pleasure, but I was unhappy. So I left the classroom and looked for pleasure elsewhere.”

**(3) If an event could be classified as related to either an emotional or a knowledge-acquisition goal; it was classified as a mixed event.**	e.g., “Today, in art class, the teacher taught us how to draw a peony. It was good to understand new things; it put us in a good mood and we gained something.”

**(4) Descriptions of some events were too simple and did not contain distinguishing information. These events were considered non-specific.**	
**Examples of these events include “Let the robot clean the house” or “Cooking meat for dinner.”**	

*SPSS 22.0 and HLM 7.0 were used in the data analysis and the significance level used in the statistical tests was set at *p* < 0.05.*

## Results

### Age Differences in Numbers of Events Related to Different Social Goals

A general description of the diary entries for the two age groups was presented (For more details, please see **Appendix**). Completion of this task took 14 days. We collected 14 diary entries from each participant. Forty-nine older adults recorded 1290 daily events in total; of these 352 and 888 were coded as events related to knowledge-acquisition and emotional goals, respectively. In addition, 26 events were related to both types of goals and classified as mixed events, and 24 events were considered non-specific because their descriptions were too simple and did not contain distinguishing information. Based on the valence rating scores for the events, 771, 197, and 276 positive, neutral, and negative events were recorded, respectively.

The total number of daily events recorded by the younger group was 1246; of these, 541 and 643 were coded as events related to knowledge-acquisition and emotional goals, respectively. In addition, 44 events were related to both types of goals and classified as mixed events, and 18 events were considered non-specific because they were too simple and did not contain distinguishing information. Based on the valence rating scores for the events, 678, 231, and 314 positive, neutral, and negative events were recorded, respectively. The descriptive statistics regarding the events related to the two types of social goals for the two age groups are shown in **Table 2**. Because the numbers of the mixed events recorded by both age groups were relatively small, they were excluded from further analysis.

**Table 2:** Descriptive statistics for events related to the two types of social goals for both age groups.

		Events related to knowledge-acquisition Goals	Events related to emotional goals
Older adults	Positive/Total	235/352 (67%)	533/888 (60%)
	Neutral/Total	71/352 (20%)	134/888 (15%)
	Negative/Total	46/352 (13%)	221/888 (25%)
Younger adults	Positive/Total	304/541 (56%)	358/643 (56%)
	Neutral/Total	123/541 (23%)	102/643 (16%)
	Negative/Total	114/541 (21%)	183/643 (28%)

The Chi-square test was performed to test the differences between the number of events related to knowledge-acquisition and emotional goals in both age groups. The Chi-square test showed significant (χ^2^ = 77.96, *p* < 0.001) differences between the numbers of events related to knowledge-acquisition and emotional goals in both age groups. Combined with the descriptive results regarding event types (see **Table 2**), these results indicated that events related to emotional goals were recorded more frequently relative to those related to knowledge-acquisition goals by both older and younger adults. This indicated that events related to emotional goals were more likely relative to those related to knowledge-acquisition goals to attract adults’ attention in daily life.

A logistic regression analysis with event type as the dependent variable (events related to knowledge-acquisition goals were used as the referential category) and age (coded as a dummy variable, 0 = younger, and 1 = older) as the predictor variable was conducted to examine the differences with respect to age in the number of events related to the two types of social goals. The results showed that event types differed significantly according to age, indicating that compared to younger adults, older adults’ events related to emotional goals occurred increasingly more frequently relative to events related to knowledge-acquisition goals [*B* = 0.75, *SE =* 0.09, Wald = 74.85, *p* < 0.001, Exp(*B*) = 2.12, 95% CI = 1.79–2.51]. The odds ratio for preference for events related to emotional goals, rather than knowledge-acquisition goals, in older adults was 2.12 times that observed for younger adults. That is, for each event related to a knowledge-acquisition goal, younger and older adults recorded 1.19 (643/541 = 1.19) and 2.51 (888/352 = 2.52) events related to emotional goals, respectively; and the number of events related to emotional goals recorded by older adults was 2.12 times that recorded by younger adults (2.52/1.19 = 2.12).

### Positivity Effect from the Perspective of Number of Events Related to the Two Types of Social Goals

The result of the positivity effect was that when compared to younger adults, older adults recorded a higher number of positive events, rather than negative events, and rated the valence of the events more positively. The average positivity index observed for the number of events related to the two social goals in each age group are shown in **Table 3**.

**Table 3:** Descriptive results regarding the positivity effect for the number of events related to knowledge-acquisition and emotional goals, and related valence scores.

	Age Group	*n*	Events related to knowledge-acquisition goals (*M* ±*SD*)	Events related to emotional goals (*M* ±*SD*)
Number	Older	49	0.66 ± 0.50	0.44 *±* 0.36
	Younger	36	0.38 ± 0.44	0.32 *±* 0.49
Valence score	Older	1227	3.80 ± 1.10	3.58 ± 1.34
	Younger	1178	3.52 ± 1.09	3.41 ± 1.30

*When the numbers of events were calculated, each participant was a case in the database, and when the valence scores were calculated, each event was a case in the database.*

From the perspective of quantity, positivity effect index was based on previous studies (Löckenhoff and Carstensen, 2007; Carstensen et al., 2011) that proposed the following equation: the positivity index = (number of positive events - number of negative events)/(number of positive events + number of negative events). The results of an independent-samples *t*-test showed that the positivity index observed for events related to knowledge-acquisition goals in older adults was larger relative to that observed for younger adults, *t*(83) = 2.66, *p* < 0.05, Cohen’s *d* = 0.59, which indicated that older adults’ preference for recording positive events, rather than negative, related to knowledge acquisition goals was greater relative to that observed for younger adults. However, the age-related positivity effect for events related to emotional goals did not differ significantly, *t*(83) = 1.28, *p* > 0.05, Cohen’s *d* = 0.28, indicating that preference for recording positive events, rather than negative, related to emotional goals was similar in both age groups.

### Positivity Effect from the Perspective of Valence Scores for Events Related to the Two Types of Social Goals

From the perspective of quality, a comparison was made for each event’s valence rating score. Each event was a case in the database to be compared that was nested in each participant. Therefore, multilevel modeling was conducted to test whether age group and event type moderate valence scores. We analyzed the data using the Hierarchical Linear Modeling program (HLM; version 7.0) that is ideally suited to modeling nested data-sets with missing data and used the following lagged models:

Level-1 Model:

Valence scores_ij_ = β_0i_ + β_1j_ (Age group_ij_) + β_2j_ (Event type_ij_) + β_3j_ (Age group^∗^Event type_ij_) + r_ij_

Level-2 Model:

β_0i_ = γ_00_ + u_0j_
β_1i_ = γ_10_
β_2i_ = γ_20_
β_3i_ = γ_30_

β_0i_ represents the intercept of age group *j*, indicating the mean value of all participants’ valence scores. β_1j_ represents the coefficient of age group *j*, indicating that when age group varied from older adults (coded as 1) to younger adults (coded as 0), the valence score varied. β_2j_ represents the coefficient of event type *j*, indicating that when event type varied from events related to knowledge-acquisition goals (coded as 0) to events related to emotional goals (coded as 1), the valence score varied. β_3j_ represents the coefficient of age group^∗^event type, indicating the predictive effect of age group^∗^event type on valence scores. *r*_ij_ indicated a residual term specific to observation *i* in unit *j*. *u*_0j_ represents the residual variation that was not explained by level-2. Level-2 represents intra-individual differences in these 14 days’ daily life events. Level-1 represents inter-individual differences in event type and valence scores.

Mixed Model:

Valence scores_ij_ = γ_00_ + γ_10_ (Age group_ij_) + γ_20_ (Event type_ij_) + γ_30_ (Age group^∗^Event type_ij_) + u_0j_ + *r*_ij_

In the empty model, σ^2^ = 1.42 and τ = 0.15 that resulted in an ICC = 0.10. This result suggested that 10% was the amount of variance at the between-person level compared to 90% at the within-person level.

When age group, event type and age group^∗^event type were entered in level-1 analysis, the results showed that age group could predict the valence scores significantly (β_10_ = 0.35, *SE* = 0.12, *t* = 3.00, *p* < 0.01, semi-partial *R*^2^ = 0.01), and event type could not predict the valence scores significantly (β_20_ = -0.06, *SE* = 0.07, *t* = -0.79, *p* > 0.05, semi-partial *R*^2^ = 0.003). The predictive effect of age group^∗^event type was not significant (β_30_ = -0.16, *SE* = 0.11, *t* = -1.53, *p* > 0.05, semi-partial *R*^2^ = 0.001). These results indicated that older adults rated the events’ valence more positively than younger adults. Moreover, the events type did not moderate the age differences in the valence scores. Thus, the age-related positivity effects were observed by using valence ratings of both types of social goals reported in the context of daily events. The average positivity index observed for valence scores in both age groups are shown in **Table 3**.

## Discussion

The study examined age differences in daily events related to social goals. Using the daily diary method, older and younger adults’ emotional experiences resulting from daily life events were compared to examine the positivity effect in the context of social goals in daily life. The results indicated that although both age groups recorded a higher number of events related to emotional goals, relative to events related to knowledge-acquisition goals, the odds ratio for emotional events related to knowledge-acquisition goals in the older group was 2.12 times that observed for the younger group. In addition, relative to younger adults, older adults showed stronger preference for recording positive events, rather than negative events, related to knowledge-acquisition goals, and they rated the events related to both knowledge-acquisition goals and emotional goals more positively. The overall results indicated that the positivity effect existed, to some extent, in the background of social goals.

### Age Differences in Events Related to Social Goals

Both older and younger adults recorded a higher number of events related to emotional goals, relative to those of events related to knowledge-acquisition goals, indicating that events related to emotional goals are more likely to capture an adult’s attention in daily life relative to those related to knowledge-acquisition goals. This is consistent with the findings of previous studies indicating that emotional events attracted participants’ attention (Carstensen et al., 2000). In addition, Rovenpor et al. (2013) showed that participants of all ages selected a significantly larger amount of emotional material relative to that of neutral material. The odds ratio for preference for events related to emotional goals, rather than knowledge-acquisition goals, observed for older adults was higher relative to that observed for younger adults. It should be noted that the finding that older adults recorded a lower number of events related to knowledge-acquisition goals, relative to that of events related to emotional goals, is understandable, as their primary tasks in daily life no longer involved learning. However, knowledge-acquisition events and emotional events were not a trade-off continuum or components of the whole; therefore, a lower number of events related to knowledge-acquisition did not necessarily indicate that a higher number of events related to emotional goals would be recorded. Therefore, the finding that the odds ratio for preference for events related to emotional goals was higher, relative to that observed for those related to knowledge-acquisition goals in the older group was consistent with their emotional goals. Younger adults also recorded a high number of events related to emotional goals relative to that recorded for events related to knowledge-acquisition goals. While it did not reflect the prioritization of their social goals, this result is consistent with the results of research examining differences in emotional material, memory, and emotional experiences between older and younger adults in which both groups exhibited memory bias favoring emotional over neutral materials, (Carstensen and Turk-Charles, 1994) and expressed subjective levels of emotional experience that were equal in intensity (Labouvie-Vief et al., 1989; Levenson et al., 1991; Lawton et al., 1992; Carstensen et al., 1997). Therefore, when there is no competition between the two types of social goals in daily life, younger adults might not prioritize knowledge-acquisition goals.

### The Positivity Effect in the Context of Different Social Goals in Older Adults

The term positivity effect in previous studies refers to an observed age-related increase in the preference for positive over negative information in attention and memory (Charles et al., 2003; Mather and Carstensen, 2003). The positivity effect in the present study mainly referred to whether older adults recorded more positive events and whether they rated events positively. This differed from general research that focused on the positivity effect for emotional well-being. From the perspective of quantity with respect to the positivity effect, the results indicated that older adults showed greater preference for recording positive events, rather than negative events, related to knowledge-acquisition goals relative to that observed in younger adults. From the perspective of quality with respect to the positivity effect, older adults’ ratings of events related both to knowledge-acquisition goals and emotional goals were more positive relative to those of younger adults. This finding indicates that older adults could have observed a higher number of positive aspects of events related to knowledge-acquisition and emotional goals.

For example, an older woman wrote the following in her diary: “Today, I went to the Aging Lab at Beijing Normal University to take part in tests. It was a very happy thing and I acquired much knowledge constantly. It’s never too late to learn.” Her rating score for the emotional experience resulting from this event that was related to knowledge acquisition was 4. In contrast, a younger woman wrote the following in her diary for a similar event: “In the afternoon, I attended the multimedia courseware class. Unfortunately, after half of the class, I found that I did not quite agree with his teaching method. Most students were helpless in such a poor-quality class. They felt they had no choice but to skip the class.” Her rating score for the emotional experience resulting from this event was 2, and was more negative relative to the rating provided by the older woman. The results of a further analysis of the events showed that, relative to younger adults, older adults emphasized their emotional satisfaction more strongly. In addition, despite experiencing a decline in their cognitive resources, older adults maintained their optimism and had lower expectations. The reason why older adults’ emotional reactions to the acquisition of knowledge were more positive, relative to those of younger adults, could have been that the older adults focused on the positive aspects of negative events (Isaacowitz et al., 2008). Therefore, although older adults prioritize emotional goals, they could also exhibit the positivity effect when confronted with knowledge-acquisition goals.

From the perspective of quantity with respect to the positivity effect, the finding indicating that there was no positivity effect for events related to emotional goals in older adults could have occurred may be because they were unlikely to experience emotions involving high levels of arousal (e.g., excitement and pride; Lawton et al., 1992). Scheibe et al. (2013) reported that compared to younger adults, older adults’ experience of emotions involving low levels of arousal (e.g., calmness, peacefulness, and relaxation) increased. Another reason may be that younger adults may record more positive emotional events, leading to a relatively larger positivity index in the younger group. Thus, there was no positivity effect from the perspective of quantity in the proportion of emotional goals. This may be because of cultural differences in goals and emotions. Chinese older adults are relatively traditional and conservative, and do not readily show high arousal, joy, and anger. Even so, the actual reasons why the perspective of quantity with respect to the positivity effect was not observed for events related to emotional goals in older adults require further clarification. In brief, the difference in the results regarding events related to knowledge-acquisition and emotional goals suggested that examination of the positivity effect in older adults in the contexts of different social goals is required.

### Limitations and Future Research Directions

The present study explored age differences in daily life events from the perspective of social goals. The results not only provided evidence to support the SST, but also indicated that relative to younger adults, older adults showed stronger preference for recording positive events related to knowledge-acquisition goals. However, there are still several limitations.

First, to determine whether the SST was relevant in older adults’ daily life events, the events were classified as events related to knowledge-acquisition or emotional goals according to the content of the participants’ diary entries. However, we did not seek to determine whether older adults’ behavioral motivation resulted from knowledge-acquisition or emotional goals. Participants were neither ask to rate how each event related to the two goals. Future studies should examine the relationship between behavioral motivation and daily life events and ask participants to provide a subjective rating for each event related to the two goals so as to make the conclusion more convincing. Moreover, the sample size was also a limitation in the present study. Future studies should involve a larger sample size which may contribute to a power effect size and more reliable conclusions.

Second, the SST emphasizes the relationship between future time perception and behavioral goals. The current results provide only an indirect indication that future time perception was related to older adults’ behavioral goals. Further research should explore the relationship between future time perception and daily life events directly, and combine future- and present-oriented goals with emotional experience. Also, future research should focus on cognitive resources and its potential relationship with the positivity effect.

Third, using actual diaries can be considered a limitation of this study. Though reminders were sent periodically to maximize compliance, we were still not able to track compliance (e.g., completing events recording on time) and this issue may have been exacerbated in older adults given that they returned the diaries after 2 weeks. In contrast, online surveys offer a major advancement in this regard. Given today’s technology, especially mobile technology, paper diaries were not the most optimal way of collecting data. If reminders were sent by email, diaries could have been presented via an online survey tool as well. Thus, diaries should be presented using modern technology in future studies.

## Ethics Statement

This study was approved by the Ethics Committee of the School of Psychology at Beijing Normal University and written informed consent was obtained from all participants.

## Author Contributions

LJ and HP designed the research, analyzed data, and wrote the manuscript. XX collected data.

## Conflict of Interest Statement

The authors declare that the research was conducted in the absence of any commercial or financial relationships that could be construed as a potential conflict of interest.

## Appendix

### Number of Events Related to Social Goals in Older Adults

Forty-nine older adults recorded 1290 daily events in total.

(1) According to the coding system, there were 352 events related to knowledge-acquisition goals (e.g., “I attempted to draw a hill. Though I did not achieve a satisfactory result, I comforted myself with the fact that I had just attended one lesson, after all. I should not be anxious about success, and I understand it comes gradually and I’ll learn it step by step.”).
(2) There were 888 events related to emotional goals (e.g., “I had lunch with Xiaochen, whom I had not seen for a long time. We were chatting speculatively while eating, and 2 h passed. Friendship is a very pleasant thing.”).
(3) There were 26 events related to both types of goals that were classified as mixed events. (e.g., “In class, the teacher teaches so carefully that I listen with interest and feel better.”).
(4) The descriptions of 24 events were too simple and did not contain distinguishing information; these events were considered non-specific (e.g., “I took a nap at noon, and then I watched TV for a while.”).

### Number of Events for Different Types of Valence in Older Adults

(1) Based on the valence rating scores, there were 771 positive events (e.g., “I watched TV and DVDs with my grandson after lunch. He talked to me joyfully while watching them.”). This event was given a score of 5 (extremely positive).
(2) There were 197 neutral events (e.g., “On 15th March, I watched some TV programs and repeatedly told myself to be as careful as possible when purchasing things, in case I buy fake products.”). This event received a score of 3 (neutral).
(3) There were 276 negative events (e.g., “I answered the phone at 10 o’clock in the morning, and I was told to go to Shuangan shopping mall to buy a water-saving machine. I said that I had no time to go there. Then, they asked me whether I could go to the shopping mall tomorrow. After I said that I would have no time the following day, they asked whether I could go the day after that. The phone call bothered me, and I had to stop talking to them.”). This event received a score of 1 (very negative).

### Number of Events Related to Social Goals in Younger Adults

The younger group recorded 1246 daily events in total.

(1) According to the coding system, there were 541 events related to knowledge-acquisition goals (e.g., “The topic of today’s reading class was very meaningful. I argued about it with my classmate for a long time. Although we failed to persuade each other, we enjoyed the process.”).
(2) There were 643 events related to emotional goals (e.g., “Finishing the morning classes meant that my curriculum for this week was almost finished, so my classmates and I went to Jinwuxing market to buy some fruit and other items. It was so much better to go around with them.”).
(3) There were 44 events related to both types of goals, and were classified as mixed events (e.g., “This afternoon, I spent time doing homework, which has been dragging on for a long time. I find that if you do things, they become simple. I taught my classmate to swim. Helping others made me feel good.”).
(4) The descriptions of 18 events were too simple and did not contain distinguishing information; these events were considered non-specific (e.g., “I went to bed early, as there was nothing to do.”).

### Number of Events for Different Types of Valence in Younger Adults

(1) Based on the valence rating scores, there were 678 positive events (e.g., “I got up early, so I had breakfast and attended classes on time. I helped my roommate get some water, so she thanked me a lot.”). This event received a score of 5 (very positive).
(2) There were 231 neutral events (e.g., “After dinner, I watched TV until 1:00 in the morning. I cannot keep myself from watching TV. I really cannot be in this state any longer.”). This event received a score of 3 (neutral).
(3) There were 314 negative events (e.g., “At noon, I did not want to go to home teaching, so I sent a message to my aunt, explaining that my clock was broken. Unexpectedly, my aunt was not angry and asked me to go to their home the following day, which made me feel guilty.”). This event received a score of 2 (more negative).
